# The *chytrid insurance hypothesis*: integrating parasitic chytrids into a biodiversity–ecosystem functioning framework for phytoplankton–zooplankton population dynamics

**DOI:** 10.1007/s00442-024-05519-w

**Published:** 2024-02-16

**Authors:** András Abonyi, Johanna Fornberg, Serena Rasconi, Robert Ptacnik, Martin J. Kainz, Kevin D. Lafferty

**Affiliations:** 1 WasserCluster Lunz—Biologische Station GmbH, Dr. Carl Kupelwieser Promenade 5, 3293 Lunz Am See, Austria; 2 MTA-ÖK Lendület “Momentum” Fluvial Ecology Research Group, Institute of Aquatic Ecology, HUN-REN Centre for Ecological Research, Karolina Street 29, 1113 Budapest, Hungary; 3 Department of Ecology, Evolution and Marine Biology, University of California, Santa Barbara, Santa Barbara, CA 93106 USA; 4 Université Savoie Mont Blanc, INRAE, CARRTEL, 74200 Thonon-Les-Bains, France; 5 Donau-Universität Krems, Dr. Karl Dorrek Straße 30, 3500 Krems, Austria; 6 U.S. Geological Survey, Western Ecological Research Center, at Marine Science Institute, UC Santa Barbara, Santa Barbara, CA 93106-6150 USA

**Keywords:** Buffer, Community assembly, Disturbance, Insurance effect, PUFA, Trophic transfer

## Abstract

In temperate lakes, eutrophication and warm temperatures can promote cyanobacteria blooms that reduce water quality and impair food-chain support. Although parasitic chytrids of phytoplankton might compete with zooplankton, they also indirectly support zooplankton populations through the “mycoloop”, which helps move energy and essential dietary molecules from inedible phytoplankton to zooplankton. Here, we consider how the mycoloop might fit into the biodiversity–ecosystem functioning (BEF) framework. BEF considers how more diverse communities can benefit ecosystem functions like zooplankton production. Chytrids are themselves part of pelagic food webs and they directly contribute to zooplankton diets through spore production and by increasing host edibility. The additional way that chytrids might support BEF is if they engage in “kill-the-winner” dynamics. In contrast to grazers, which result in “eat-the-edible” dynamics, kill-the-winner dynamics can occur for host-specific infectious diseases that control the abundance of dominant (in this case inedible) hosts and thus limit the competitive exclusion of poorer (in this case edible) competitors. Thus, if phytoplankton diversity provides functions, and chytrids support algal diversity, chytrids could indirectly favour edible phytoplankton. All three mechanisms are linked to diversity and therefore provide some “insurance” for zooplankton production against the impacts of eutrophication and warming. In our perspective piece, we explore evidence for the *chytrid insurance hypothesis*, identify exceptions and knowledge gaps, and outline future research directions.

## Introduction

The Irish Potato famine is reminder of what can happen when a population relies on a narrow diet. In lakes, generalist grazers like *Daphnia* push the phytoplankton community towards a low-diversity state dominated by inedible species at the expense of food-chain support for zooplankton. Indeed, the theory of biodiversity–ecosystem functioning ascribes several positive benefits to diverse communities (Table 1). A proposed source of food during phytoplankton blooms is the microbial loop (Pomeroy 1974) that occurs when bacteria, phagotrophic flagellates, and protozoans decompose organic material and then become a supplemental food source for zooplankton (Porter 1996). However, microbes are not always a sufficient replacement diet for edible phytoplankton (Taipale et al. 2012).

**Table 1:** Summary of key terms in describing the *chytrid insurance hypothesis*

Terms	Description
Additive communities	Community abundance jointly increases with species richness. Risk for disease, particularly by generalist pathogens, maximises in species-rich communities (Weis et al. 2007; and additional references therein)
Biodiversity-ecosystem functioning (BEF) theorem	A positive relationship between biodiversity and ecosystem functioning. As the number of species in an ecosystem increases, the overall functioning and stability of the ecosystem also increase due to sampling or complementarity effects (Loreau & Hector 2001)
Buffering effect	The capacity of a system, or member of the system, to lessen the effects of disturbances or changes, contributing to the overall stability and resilience of ecosystems. Buffers protect against external pressures, such as low resource availability
Insurance effect	For ecosystem functioning, insurance refers to the phenomenon where biodiversity contributes to the stability and resilience of an ecosystem. Higher biodiversity can provide insurance, ensuring that if changing conditions negatively impact some species, other species still fulfil critical ecological functions (Yachi and Loreau 1999)
Kill-the-winner (KTW) hypothesis	Density-dependent transmission increases disease prevalence in abundant host species, which results in negative density dependence and increased coexistence (Thingstad et al.,^2000^)
Mycoloop	Chytrid zoospores are highly palatable to zooplankton, offering an alternative trophic pathway that bridges the gap between inedible phytoplankton and zooplankton (Kagami et al. 2007a; Kagami et al. 2007b; Grami et al. 2011). The mycoloop (Kagami et al. 2014) facilitates energy and nutrient transfer within aquatic food webs
Nutritional quality	The biochemical composition of prey in terms of essential dietary molecules, such as sterols (Martin-Creuzburg et al. 2008) and polyunsaturated fatty acids (PUFAs) (Arts et al. 2009; Pilecky et al. 2021)
PUFA	Polyunsaturated fatty acids (PUFAs) lack multiple hydrogen atoms and contain multiple double bonds in their carbon chains. Long-chain PUFAs particularly benefit zooplankton fitness (Gulati and Demott 1997; Ruess and Müller-Navarra 2019), including the omega-3 fatty acid EPA (20:5n-3) and the omega-6 fatty acid ARA (20:4n-6) (Müller-Navarra et al. 2000; Ilić et al. 2021)
Sampling effect	In diverse communities, species that exhibit strong positive effects on ecosystem functioning are more likely to be included by chance (Loreau and Hector 2001)
Substitutive communities	Total community abundance is fixed with richness, so that high diversity communities have fewer individuals per species than low-diversity communities. Thus, host-specific infectious diseases are less likely in species-rich communities via the dilution effect (Weis et al. 2007; further references therein)
Trophic upgrading	Predators enrich their biochemical composition compared to their prey (Bec et al. 2006, 2010; Breteler et al. 1999) with essential molecules, such as sterols or long-chain polyunsaturated fatty acids (Gerphagnon et al. 2019; Rasconi et al. 2020; Veloza et al. 2006)

Like the microbial loop hypothesis, the mycoloop hypothesis aims to explain how zooplankton often persist under bloom conditions (Kagami et al. 2007a; Kagami et al. 2007b; Grami et al. 2011; Table 1). In contrast to the microbial loop, the chytrid fungi (Phylum Chytridiomycota) that infect and kill phytoplankton (not the distantly related chytrids that infect amphibian hosts), form an energetic loop that redirects energy from inedible phytoplankton to zooplankton in the form of edible fungal zoospores (Kagami et al. 2014; Frenken et al. 2017). This mycoloop provides essential dietary molecules (Taube et al. 2019; Gerphagnon et al. 2019; Rasconi et al. 2020) that benefit zooplankton (Agha et al. 2016; Frenken et al. 2020a; Frenken et al. 2020b; Abonyi et al. 2023) and exceed the carbon cycled through the microbial loop (Abonyi et al. 2023). Therefore, the mycoloop is a potentially more important source of food during bloom conditions than the microbial loop (e.g. Kagami et al. 2007a; Kagami et al. 2007b; Agha et al. 2016; Abonyi et al. 2023). But if and how chytrids contribute to pelagic food webs outside bloom events is unclear. In this perspective piece, we consider a new idea called the “*chytrid insurance hypothesis*” that considers a role for parasites in biodiversity–ecosystem functioning.

The biodiversity–ecosystem functioning hypothesis (BEF) argues that diversity can promote stability, production, and other ecosystem attributes (Naeem and Li 1997; Hooper et al. 2005; Duffy et al. 2017). This can occur if having more species in a community makes it easier for the community to respond to changing or unpredictable conditions due to mechanisms like complementarity (species play different roles) or a portfolio/sampling effect (if there are many species, at least one is bound to be successful at a particular moment) (Loreau and Hector 2001; Table 1). To that end, according to the insurance hypothesis, biodiversity increases the chance that some species maintain functioning even if others fail (Yachi and Loreau 1999; Table 1). Just as a diverse stock portfolio gives some insurance that a worker’s retirement account makes steady gains even in an uncertain financial market, biodiversity can help insure that an ecosystem will function and provide benefits even in unstable or unpredictable conditions. Thus, it seems plausible that a more diverse diet could help insure zooplankton against population crashes (Striebel et al. 2009). As envisioned by Frainer et al. (2018), there are three ways that chytrid parasites might mediate biodiversity–ecosystem functioning: (1) parasites produce edible stages, (2) infected hosts are altered in a way that adds trait diversity to a host population, (3) parasites help maintain host diversity. We propose that chytrids can do all three: (1) free-swimming chytrid zoospores are nutritious food for zooplankton, (2) chytrid sporangia can make inedible hosts edible by infection of the host, (3) chytrid host specificity maintains edible phytoplankton diversity. If true, chytrids provide insurance to zooplankton against conditions that would otherwise lead to blooms of inedible phytoplankton.

## Zooplankton and phytoplankton communities

**Fig. 1:**
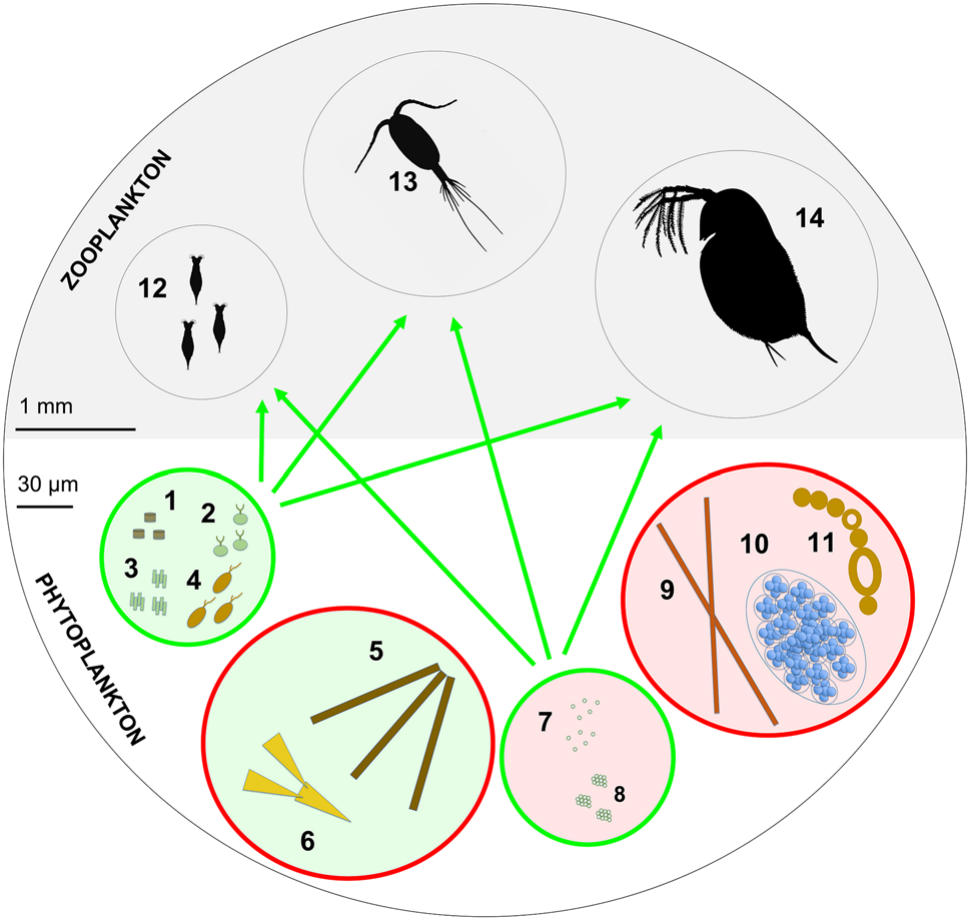
Phytoplankton vary in size and nutritional quality for zooplankton. High-quality phytoplankton are rich in polyunsaturated fatty acids (PUFA) (green background), such as the small-sized palatable *Stephanodiscus* (1), *Scenedesmus* (2), *Chlamydomonas* (3) or *Cryptomonas* (4), or, the large-sized inedible *Asterionella* (5) and *Dinobryon* (6). Poor-quality phytoplankton lack PUFA and sterols (red background), such as the small-sized edible cyanobacterium *Synechococcus* (7), the small colonial *Aphanocapsa* (8), and the inedible bloom-forming cyanobacteria *Planktothrix* (9), *Microcystis* (10) or *Dolichospermum* (11). Rotifers preferentially take up food < 10 µm (e.g. Bdelloidea, 12), whereas copepods (e.g. *Cyclops*, 13) and cladocerans (e.g. *Daphnia*, 14) eat things < 30 µm. Green arrows show dietary pathways between edible phytoplankton and zooplankton. Figures 1–11: authors’ drawings, Figs. 12–14: www.phylopic.org

Phytoplankton community composition can regulate zooplankton growth (Behl and Stibor 2015). Zooplankton can be picky eaters, preferring small- to medium-sized diet items, such as Cryptophytes and green algae (Fig. 1). Filter-feeding zooplankton, like rotifers, selectively graze on small things < 10 µm (Arndt 1993), whereas non-selective cladocerans (*Daphnia*; Burns 1968; Carpenter et al. 1993) and copepods (Vanderploeg et al. 1984; Bern 1994) eat small- and medium-sized food (< 30 μm). However, most zooplankton are unable to eat large phytoplankton (> 30 µm). Diverse phytoplankton communities tend to have more edible species. More diverse communities are also better food, because they have more lipids (Stockenreiter et al. 2012), including long-chain polyunsaturated fatty acids (LC PUFA; Marzetz et al. 2017) that zooplankton need (Tessier and Goulden 1982), but cannot synthesise (Gulati and Demott 1997; Table 1). Under eutrophication and warming conditions (Paerl and Huisman 2008; O’Neil et al. 2012), the diverse phytoplankton community often shifts from diatoms and chrysophytes to a few green algae or cyanobacteria (Ptacnik et al. 2008; Glibert et al. 2018; Gobbler 2020). Figure 1 illustrates how this compositional shift reduces the availability of LC PUFA and sterols for zooplankton (Taipale et al. 2019; Calderini et al. 2023), thereby decreasing zooplankton production (Elert et al. 2003; Ger et al. 2016; Peltomaa et al. 2017). Indeed, because phytoplankton vary in their edibility and nutritional value (Taipale et al. 2013), phytoplankton community structure can affect zooplankton production more than phytoplankton abundance (Calderini et al. 2023).

## Parasitic chytrids are pervasive and diverse in pelagic food webs

Key to our hypothesis is that chytrids are diverse and their abundance scales with host density. There are many chytrid species, and collectively, they often reach 20% and can reach almost 100% prevalence during phytoplankton blooms (Rasconi 2012). Free-swimming zoospores (~ 2–5 µm; Sparrow 1960) seek out and, depending on the chytrid species, infect a range of algae and cyanobacteria, mainly inedible taxa (Sparrow 1960; Sime-Ngando 2012; Money 2016). Upon successful infection, chytrids develop sessile sporangia that convert host biomass into spores. This reduces phytoplankton fitness and can be fatal (Frenken et al. 2017).

## Chytrid zoospores are nutritious food for zooplankton

The mycoloop is an example of how adding parasite species can directly increase ecosystem functioning. Although it might seem as if chytrids simply compete with zooplankton for phytoplankton, zoospores are eaten by both selective (e.g. rotifers, see Frenken et al. 2018) and non-selective filter feeders (e.g. cladocerans, see Kagami et al. 2007a; Kagami et al. 2007b; Agha et al. 2016). Notably, zoospores are nutritious, because they convert short-chain to long-chain PUFA (Taube et al. 2019; Rasconi et al. 2020) and can produce sterols de novo (Gerphagnon et al. 2019). Consequently, chytrids benefit zooplankton by converting inedible poor-quality phytoplankton into nutritious edible zoospores through “trophic upgrading” (Breteler et al. 1999; Veloza et al. 2006; Bec et al. 2006, 2010; Table 1).

The mycoloop hypothesis has been subject to population modelling to evaluate scenarios and explore population dynamics (Grami et al. 2011; Miki et al. 2011; Kagami et al. 2014; Frenken et al. 2020a; Frenken et al. 2020b; Thongthaisong et al. 2022). In a pioneering paper, Miki et al. (2011) found that although the mycoloop could have positive effects on zooplankton production, the overall impact could be negative if zooplankton growth efficiency was lower when feeding on chytrids than on small algae (Fig. 4, left panel). In more recent models, parasitism by chytrids was found to alleviate competition amongst edible phytoplankton, increasing zooplankton production (Kagami et al. 2014). High zooplankton densities can also reduce chytrid transmission (through consuming free-living infective zoospores) and overgraze edible algae, favouring the growth of inedible phytoplankton that can then feedback to depress the zooplankton population (Kagami et al. 2014; Thongthaisong et al. 2022). Although modelling indicates that the mycoloop predominantly influences blooms (Thongthaisong et al. 2022), this might relate to modelling assumptions that nutrients are not recycled in the system through zooplankton excretion and death. Model outcomes, thus, may hinge on critical details about how the mycoloop system is defined mathematically.

## Chytrid infection helps make inedible hosts edible

**Fig. 2:**
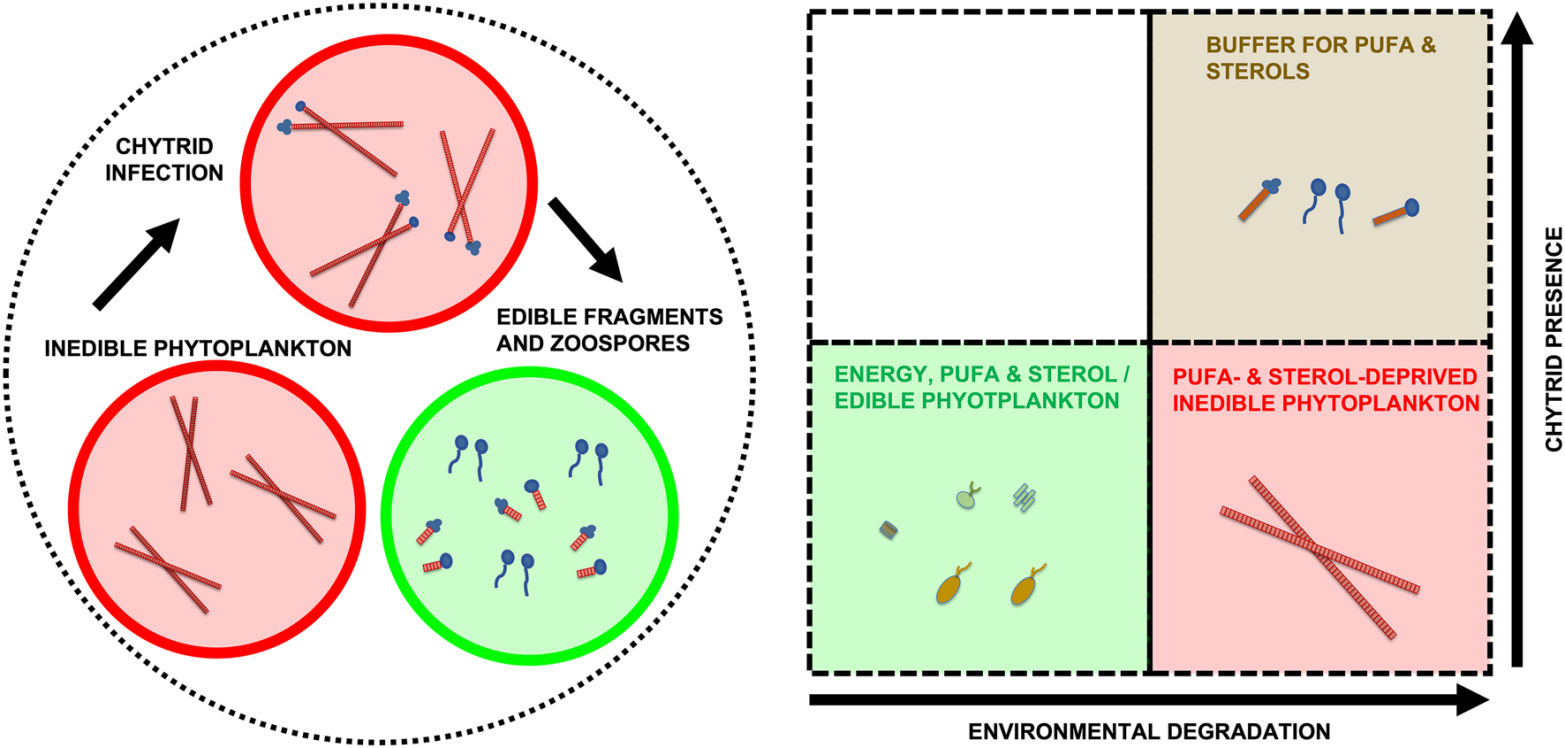
Eutrophication and warming stimulate blooms of inedible cyanobacteria that lack polyunsaturated fatty acids and sterols. Chytrid fungal parasites primarily infect inedible phytoplankton hosts and facilitate fragmentation, leading to enhanced feeding, including the consumption of edible chytrid zoospores (left). This increased availability of dietary options improves diet quality regarding PUFA and sterols. The PUFA and sterols derived from chytrids act as a buffer, supporting zooplankton nutrition during cyanobacteria blooms (right)

In addition to producing edible spores, chytrids diversify zooplankton diets by fragmenting inedible phytoplankton (Gerphagnon et al. 2013; Agha et al. 2016; Frenken et al. 2020a; Frenken et al. 2020b) and increasing their nutritional quality (Gerphagnon et al. 2019; Taube et al. 2019; Rasconi et al. 2020; Table 1). Following fragmentation, zooplankton can graze on both chytrid zoospores (Gerphagnon et al. 2019; Taube et al. 2019; Rasconi et al. 2020) and phytoplankton fragments (Abonyi et al. 2023), providing a more diverse resource to sustain zooplankton populations. By providing carbon, LC PUFA, and sterols, chytrids may act as a buffer for energetic and essential molecule requirements when they are most needed by zooplankton (Fig. 2; Table 1).

## Chytrid host specificity maintains edible phytoplankton diversity

Although it might seem like phytoplankton production alone should drive zooplankton growth rates, fast-growing edible algae in diverse communities can also enhance zooplankton growth (Marzetz et al. 2017). Zooplankton growth appears to increase because higher diversity enhances diet quality (Marzetz et al. 2017) due to a higher likelihood of encountering high-quality resources (i.e. the sampling effect; Naeem and Wright 2003). When ecosystem functions like zooplankton production and water quality are supported by phytoplankton diversity, there is the potential for a positive BEF relationship (Fridley 2001; Loreau and Hector 2001). Indeed, a positive BEF relationship is a key assumption on which the *chytrid insurance hypothesis* rests. Specifically, chytrids may diversify phytoplankton communities with complementarity or portfolio effects that benefit zooplankton communities as well as physical processes like nutrient cycling in water.

**Fig. 3:**
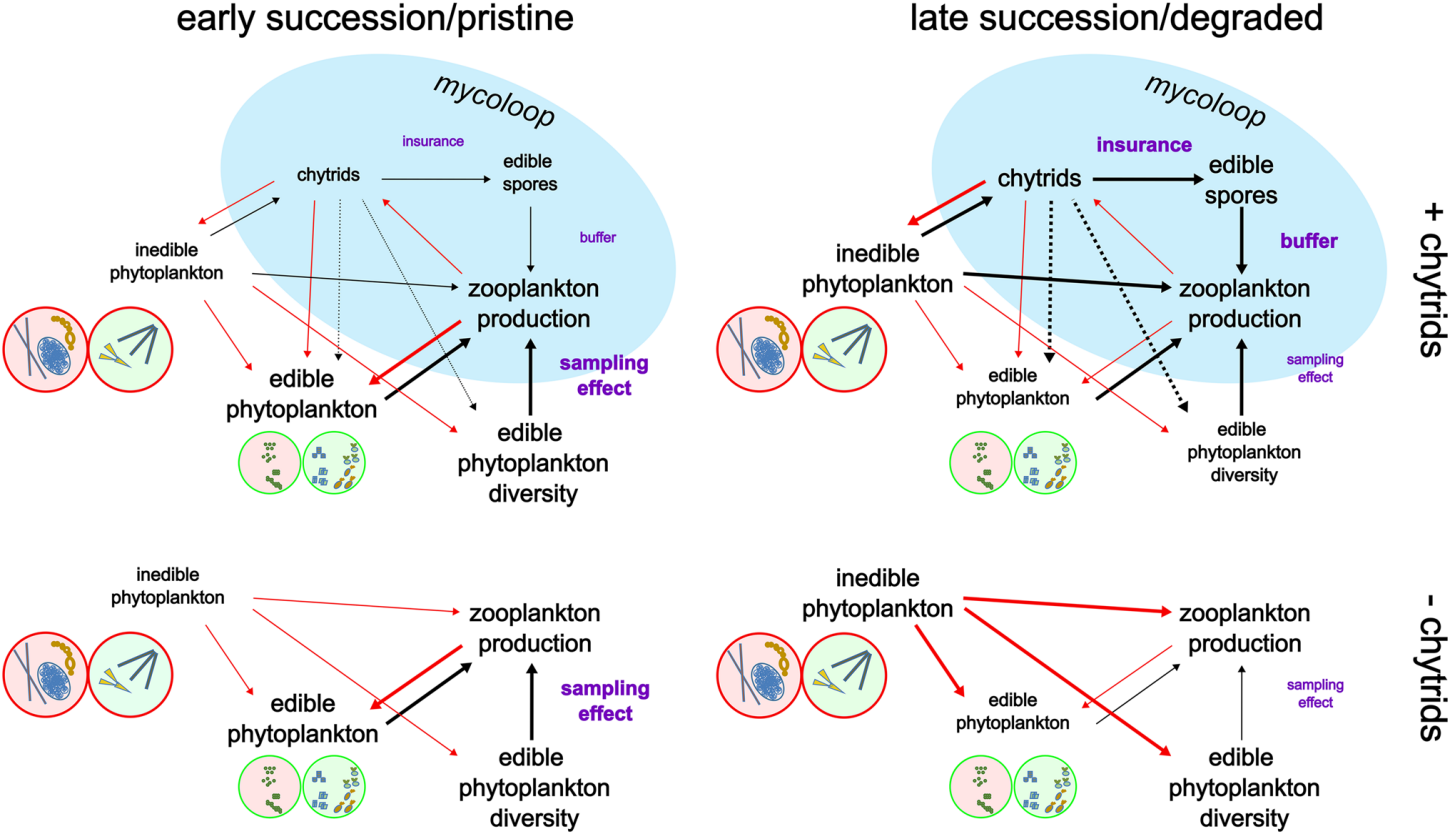
Edible phytoplankton serve as vital sources of energy and essential dietary molecules for zooplankton during early successional stages. A high diversity of edible items enhances zooplankton biomass production and increases the likelihood of encountering high-quality diet items (bottom left, sampling effect). However, as succession or degradation occurs, inedible phytoplankton become more prevalent, hindering edible phytoplankton and zooplankton production (bottom right, weaker sampling effect). Chytrids take advantage of inedible phytoplankton dominance and provide an alternative pathway to buffer energy and essential molecules through zoospore production in the mycoloop and increased feeding (top left, chytrid insurance). Furthermore, chytrids can suppress inedible phytoplankton, indirectly benefiting edible phytoplankton and their diversity (dashed lines). Environmental degradation amplifies inedible phytoplankton blooms, thereby augmenting the buffering effects that chytrids can provide (top right, increased chytrid insurance). Please refer to the colour codes of phytoplankton in Fig. 1. Black arrows represent positive interactions, red arrows represent negative interactions, and dashed arrows represent indirect interactions. Arrow width indicates relative interaction strength between each scenario

Succession in plankton can be predictable, with some systems experiencing seasonal blooms (Sommer et al. 1986). In early stages, phytoplankton density is low, and reduced competition allows additive community assembly (see Table 1), promoting diverse and rapidly growing communities (Weis et al. 2007). Edible phytoplankton are expected to particularly benefit zooplankton during early successional stages (Thongthaisong et al. 2022). In non-bloom conditions, chytrid prevalence typically remains moderate (ranging between 3 and 20%, Sime-Ngando 2012; Gsell et al. 2022). However, because most chytrids are host specific (e.g. *Rhizophydium planktonicum, Rhizophydium megarrhizum*; Frenken et al. 2017 and references therein), “kill-the-winner” dynamics, where numerically common taxa suffer more from parasitism (Thingstad et al. 2000; Table 1), might maintain diversity amongst edible phytoplankton. Indeed, the community composition of chytrids often follows the seasonal dynamics of the phytoplankton community, with infection rates reflecting phytoplankton composition and abundance (Rasconi et al. 2012). Thus, the interactions amongst chytrids, phytoplankton and zooplankton may vary considerably over succession (Fig. 3).

At later successional stages, zooplankton depress the abundance of edible phytoplankton, which releases nutrients and may allow inedible phytoplankton to undergo monospecific blooms (Huisman et al. 1999; Prince et al. 2008; Chakraborty and Feudel 2014). Depending on the presence of generalist grazers like *Daphnia* (Tessier and Woodruff 2002) and the lake’s trophic state, large-sized diatoms, green algae, or cyanobacteria can eventually dominate over others (Sommer et al. 1986). Collectively, eutrophication, grazing on edible phytoplankton, and lake warming intensify the dominance of inedible phytoplankton, particularly cyanobacteria (Paerl and Huisman 2008; O’Neil et al. 2012), resulting in more efficient resource use for phytoplankton but impaired ecosystem functioning for zooplankton (Filstrup et al. 2014). Like bloom-forming taxa, chytrid densities increase with lake trophic state and track positively with host dominance (Rasconi et al. 2012; Thongthaisong et al. 2022). High rates of chytrid infection can suppress host density (Agha et al. 2016; Frenken et al. 2018; 2020a; 2020b; Abonyi et al. 2023), suggesting a strong regulatory role for chytrids under bloom conditions. By predominantly infecting most inedible phytoplankton (Van Donk and Bruning 1992; Frenken et al. 2017; Sassenhagen et al. 2023), chytrids might reduce the rate at which inedible species outcompete edible species (Rasconi et al. 2011). For instance, phytoplankton diversity increased after a chytrid-induced decline of the dominant inedible diatom, *Asterionella formosa* (Canter and Lund 1951; Van Donk and Ringelberg 1983), remaining the best evidence that chytrids can foster phytoplankton diversity.

Although the chytrid insurance hypothesis assumes that chytrids increase phytoplankton community evenness and reduce phytoplankton abundance, the diversity and abundance of phytoplankton communities (and the relationship between diversity and abundance) should feedback to affect chytrid success. Such feedbacks may strengthen or weaken the *chytrid insurance hypothesis.* For instance, if phytoplankton communities are additive (more nutrients or diverse habitats leads to more phytoplankton individuals per species), then host-specific and generalist chytrids should increase in prevalence with phytoplankton diversity, further increasing phytoplankton evenness (and chytrid insurance) whilst regulating phytoplankton abundance. If phytoplankton communities are substitutive (more diversity leads to fewer individuals per species; Table 1), then increased phytoplankton diversity should not affect overall chytrid prevalence, but should increase the proportion of generalist chytrid species, thereby reducing the potential for kill-the-winner dynamics and chytrid insurance. Furthermore, if zoospores do not discriminate amongst host species when attempting to infect, a diversity-dilution effect could result (Frenken et al. 2017) whereby increased phytoplankton diversity would decrease host-specific chytrid species, further reducing chytrid insurance. Finally, phytoplankton diversity-abundance relationships might change with succession or environmental conditions, making chytrid insurance context dependent.

## Testing the chytrid insurance hypothesis

Field observations, lab experiments, and mathematical models have been used to investigate the mycoloop hypothesis and these approaches could also be used to assess the conditions under which the chytrid insurance hypothesis might hold. For instance, field observations on the mycoloop could be expanded to include more information about the associations between phytoplankton diversity, chytrid prevalence, and zooplankton growth and abundance. Obvious predictions are that zooplankton growth rates increase with phytoplankton diversity and that chytrids are more likely to infect abundant phytoplankton species. One growing source of information is environmental DNA samples that can indicate chytrid and phytoplankton diversity, albeit with limitations related to the paucity of sequence data for parasitic chytrids and the inability to quantify abundance (Frenken et al. 2017). This and more traditional information are needed to document when the *chytrid insurance hypothesis* is important in nature.

Some investigators have manipulated the presence of chytrids in microcosms to test cause and effect relationships. Chytrids can be isolated from field collections and kept in culture for short-term microcosm experiments (e.g. Agha et al. 2016; Kagami et al. 2007a; Kagami et al. 2007b; Abonyi et al. 2023). Future work could manipulate phytoplankton diversity to evaluate the conditions under which chytrids can increase zooplankton growth. Furthermore, infection experiments between different chytrid and phytoplankton species would help evaluate the key assumption that chytrids engage in kill-the-winner strategies that promote phytoplankton diversity. It should also be possible to gauge how diet quality and diversity affect zooplankton growth in synthetic communities. Finally, by manipulating the presence or absence of chytrids, researchers could assess the degree that the mycoloop fosters phytoplankton diversity and delays or constrains succession towards blooms and associated zooplankton crashes. Given that there are many potential reasons for associations between chytrid parasitism, phytoplankton diversity, and zooplankton growth rates, laboratory experiments will be key to deciphering whether chytrids help foster a positive biodiversity–ecosystem functioning relationship or are simply correlated with it.

**Fig. 4:**
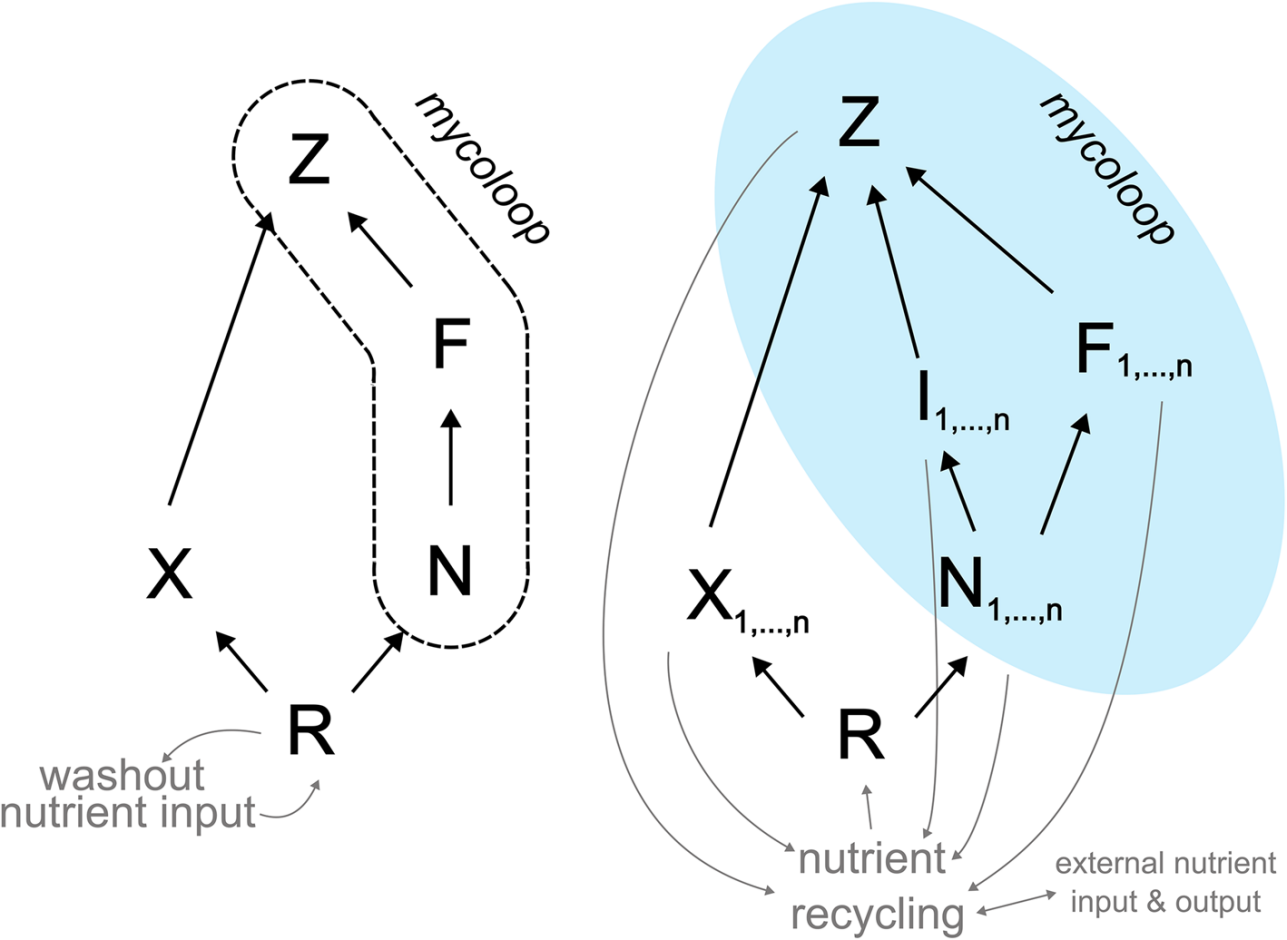
Theoretical NPZ model proposed by Miki et al. (2011), Kagami et al. (2014) and Thongthaisong et al. (2022) (left); and extension including the insurance effects of chytrids (right). Resources (R) such as dissolved nutrients support populations of *n* species of edible (X_1,…,n_) and inedible (N_1,…,n_) phytoplankton. Zooplankton (Z) feed on edible phytoplankton, as well as on *n* species of fungal chytrid zoospores (F _1,…,n_), i.e. the “F-Z feeding link”. Species of infected inedible phytoplankton (I_1,…,n_) become palatable to zooplankton with time

The *chytrid insurance hypothesis* could also be subject to population modelling to evaluate scenarios and explore population dynamics difficult to evaluate empirically. Models could be designed to determine the parameter ranges under which insurance occurs and the extent and nature to which insurance affects pelagic food webs. Doing so is not trivial because to identify the range of conditions under which the presence of chytrids facilitates a positive BEF relationship would require modelling food diversity, food quality, and phytoplankton diversity. Although such models have not been attempted, foundational mycoloop models have fallen into two categories: Lotka–Volterra style NPZ models (see Franks 2002) like Miki et al. (2011; Fig. 4, left panel) and food-web models as pioneered by Grami et al. (2011). Food-web models may be better suited for encompassing a broader diversity of edible and inedible phytoplankton and their various indirect effects. Alternatively, Smith et al. (2016) provides a model of phytoplankton diversity designed to evaluate BEF that might be altered to include chytrids. Such models are not simple, but seem worth exploring.

Regardless of the modelling framework, considering that chytrids have distinct parasitic and dispersal stages might lead to different dynamics than the current modelling approach of treating chytrid spores like predators (Miki et al. 2011; Thongthaisong et al. 2022). This is because recognising an infected class for phytoplankton will allow chytrids to directly compete with uninfected hosts for resources, and suffer the consequences of intimacy if hosts die (Fig. 4, right panel). Indeed, models could benefit from more information about the chytrid life cycle, such as which hosts are infected by spores, and the effect of sporangia on infected host reproduction and mortality. Another consideration for modelling is that chytrid resting stages may increase system stability or that environmental factors like light and temperature can constrain infection dynamics (Gsell et al. 2022). Finally, allowing nutrient recycling through zooplankton excretion and death (Barranco et al. 2020) would reduce phytoplankton competition for nutrients, and this might alter previous modelling conclusions that chytrid effects are limited to bloom periods (Fig. 4, right panel). These suggestions should be approached with the understanding that incorporating additional complexities in models can reduce tractability and pose challenges in parameterisation. Yet when combined with field and experimental work, modelling will likely be needed to fully understand the conditions under which the *chytrid insurance hypothesis* applies.

## Conclusion

In posing the *chytrid insurance hypothesis*, we integrate the mycoloop into a biodiversity–ecosystem functioning framework. Biodiversity might benefit zooplankton production in three ways. First, adding diversity in the form of edible chytrid parasites creates potential for the mycoloop to benefit zooplankton directly. Second, chytrid parasites further add to diet diversity by altering the edibility of infected hosts. Finally, if phytoplankton diversity leads to more predictable and higher quality food, then kill-the-winner dynamics that promote phytoplankton diversity could reduce the conditions under which non-edible phytoplankton species bloom. All of these outcomes seem plausible, at least under some conditions, but models, experiments, and observations are needed to test core assumptions and refine predictions.

## Data Availability

No data are included in the manuscript.
